# Compression–tension cell with sample manipulator for *in situ* X-ray nanotomography experiments

**DOI:** 10.1107/S1600577525005053

**Published:** 2025-07-14

**Authors:** Arun J. Bhattacharjee, Harold S. Barnard, Alastair MacDowell, Dilworth Parkinson, Harrison P. Lisabeth

**Affiliations:** a Energy Geosciences Division Lawrence Berkeley National Laboratory Berkeley CA94720 USA; b Advanced Light Source Lawrence Berkeley National Laboratory Berkeley CA94720 USA; ESRF – The European Synchrotron, France

**Keywords:** X-ray tomography, compression–tension cell, sample manipulator, mechanical testing, fluid flow-through

## Abstract

*In situ*X-ray nanotomography experiments where tensile or compressive force is applied on the sample require specialized equipment. A compression–tension device with fluid flow-through capability has been designed for X-ray nano­tomography beamlines. The compression–tension cell is equipped with a triaxial stage for sample alignment and a high sensitivity loadcell for measurement of applied force. To handle the <100 µm samples used for X-ray nanotomography imaging and for loading samples on the compression–tension cell a sample manipulator has been built. The sample manipulator is capable of selecting a single <100 µm particle for nanotomography scanning while viewing multiple samples under an optical microscope. To test the functionality of these two devices an initial compression experiment involving two glass beads was performed. To demonstrate instrument stability two spherical glass beads were compressed from a no load condition until one of the beads fractured. Nanotomography data were collected at each step of increasing compressive force. The experimentally observed contact area of the spherical glass beads was compared with the theoretical estimate using the Hertz analysis. To demonstrate the fluid flow capability, two calcite grains were compressed against each other under a calcite saturated solution. Surface topological changes were observed for the stressed grain contact area.

## Abstract 2

An environmental cell with fluid flow-through capability for performing *in situ* nano-mechanical experiments at X-ray nanotomography beamlines and a sample manipulator for mounting nanotomography samples are described.

## 1. Introduction

3D characterization is the analysis that reveals the properties of a sample in all three dimensions, *i.e.* length, breadth and height. There are several types of 3D characterizations that have been developed over the years which evaluate and capture different properties in a sample. Some of these are destructive in nature where the sample is sectioned or partially destroyed to access the interior of the sample. Other techniques are non-destructive where the interior of the sample can be accessed without destroying the sample. Application of either destructive or non-destructive 3D characterization depends on the nature of the properties of the samples that are being evaluated.

First knowledge of a sample is visual where the sample surface is seen under ambient light or under an optical microscope. This is the genesis of the field of imaging which answers the very fundamental question of what the sample looks like. Analyzing and understanding the features of the sample with higher resolution than what is seen by the naked human eye is the microstructural characterization of the sample. Microstructural characterization can be performed with a wide range of resolutions starting with optical light microscopy at the lower resolution, and atom probe tomography (Miller *et al.*, 2012 ▸; Kelly & Miller, 2007 ▸; Müller *et al.*, 1968 ▸) and transmission electron microscopy (Liang *et al.*, 2017 ▸; Williams & Carter, 1996 ▸; Zhou *et al.*, 2022 ▸) at the higher resolutions. These imaging techniques are inherently 2D in nature but can be made a 3D imaging technique by capturing multiple images of the sample at different cross-sections with uniform spacing. This in turn requires repeated destructive sectioning of the sample and each section 2D imaged using, for example, optical microscopy (Engelkes *et al.*, 2018 ▸) and/or scanning electron microscopy (Maire *et al.*, 2001 ▸). When these cross sections are stacked, the microstructural data become 3D. This process involves extensive sample preparation using mechanical polishing, microtome or focused ion beam sectioning. Such imaging techniques are acceptable when the integrity of the sample post 3D imaging is not required. However, destructive 3D imaging techniques cannot be applied for capturing dynamic changes in a sample. This requires non-destructive 3D imaging techniques such as X-ray tomography. X-ray tomography does not require extensive sample preparation to provide 3D microstructural data. In this technique 2D radiographs are collected as the sample is rotated 180° or 360° (Maire *et al.*, 2001 ▸). The collected radiographs are then processed using reconstruction algorithms to obtain a series of 2D reconstructed images that when stacked form the 3D data microstructural data of the sample (Barnard *et al.*, 2018 ▸). To perform time resolved or *in situ* experiments where the sample is subjected to desired physical and chemical conditions, imaging is performed at different time steps to capture the dynamic changes of the sample in 3D (Bhattacharjee *et al.*, 2021 ▸). This 3D imaging with time dependance is also known as 4D imaging.

Specialized equipment is required to create the physical and chemical conditions under which *in situ*X-ray tomography is performed. This can be a furnace capable of achieving the desired temperature (Barnard *et al.*, 2018 ▸; Antonelli *et al.*, 2020 ▸; Kudrna Prašek *et al.*, 2018 ▸) or a compression–tension cell capable of achieving the desired force (Ruf *et al.*, 2023 ▸; Philippe *et al.*, 2016 ▸; Voltolini *et al.*, 2019 ▸). Construction of these *in situ* environmental cells can vary from laboratory based X-ray tomography to synchrotron X-ray tomography. The common parameters that are taken into account during the construction of these devices are: (i) the size and weight of the equipment, as the equipment needs to fit in between the optical components of the tomography system and the overall weight of the equipment should be within the weight limit of the tomography stage; (ii) the construction material of the equipment, which should allow sufficient X-ray transmission for imaging of the sample and (iii) the environmental cell, which should be capable of achieving the desired physical and chemical conditions.

For synchrotron X-ray microtomography several environmental cells have been successfully developed and deployed over the last couple of decades (Barnard *et al.*, 2018 ▸; Antonelli *et al.*, 2020 ▸; Kudrna Prašek *et al.*, 2018 ▸). These *in situ* experimental cells are capable of performing experiments at multiple elevated temperatures and pressures. However, a fundamental challenge in microtomography is the resolution limit. Microtomography instruments usually consist of imaging 2D transmission radiographs of the sample via a single crystal of X-ray scintillator material. X-ray and electron scattering within the sample and scintillator limit the spatial resolution to ∼0.5 to 1 µm depending on the setup. When imaging resolution higher than that of microtomography is desired, X-ray nanotomography can be used. This technique uses Fresnel zone plates that act as an X-ray lens to magnify the 2D transmission radiograph of the sample (Gürsoy *et al.*, 2014 ▸). The Fresnel optics puts constraints on the field of view (FOV) and beam energy used for imaging. These constraints in turn are translated to the design of the environmental cells for *in situ* nanotomography experiments.

The FOV of nanotomography is several times smaller than that of microtomography and generally ranges from 20 to 100 µm and has a depth of focus (DOF) of <100 µm. A smaller DOF also restricts the size of the sample that can be imaged. The largest sample size that a nanotomography setup with 100 µm × 100 µm FOV can accommodate is 80–100 µm. Such samples are barely visible with the naked eye and are extremely challenging to mount onto sample holders and *in situ* experimental cells with free hands.

The high imaging resolution of the nanotomography systems also requires specialized environmental cells (Kaira *et al.*, 2019 ▸). Experiments probing the mechanical properties of a sample requires high sensitivity load measurements as the applied pressure on a sample is a function of the contact area of the sample, which is small for nanotomography samples. Thermal sensitivity of the sample and the environmental cell is another important component in these experiments. As nanotomography has high imaging resolution, small sample movements due to temperature variations in and around the sample cause imaging artifacts in the reconstructed images and need to be avoided. Also, in the case of experiments where the interfacial interaction of two samples under pressure is analyzed, a small tilt in either of the samples causes difficulty in sample alignment and reconstruction.

Addressing these problems, we have constructed a compression–tension cell with fluid flow-through capability which is specifically designed for usage in X-ray nanotomography systems to perform *in situ* compression–tension experiments. To solve the problem of mounting samples in the cell we have constructed a sample manipulator. In this paper we describe the construction and operating principle of both devices with some preliminary experimental results.

## 2. X-ray nanotomography sample manipulator system

The FOV for X-ray nanotomography is small (20–100 µm) which requires the sample being scanned to be small as well. Samples <100 µm are extremely difficult to handle by hand. Additionally, nanotomography samples are attached to the tip of a pin as anything larger can obstruct the sample from the beam due to slight tilt of the sample holding pin tip. To address this issue we have designed a sample manipulator that can be attached to an optical microscope. This allows the viewing of a range of small sample particles, from which the desired particle is chosen and extracted by the needle of the manipulator and attached to the tip of the tomography mounting pin, which is then placed within the *in situ* rig, which in turn is mounted on the rotating stage of the nanotomography system for imaging.

### 2.1. Sample manipulator – description

**Figure 1:**
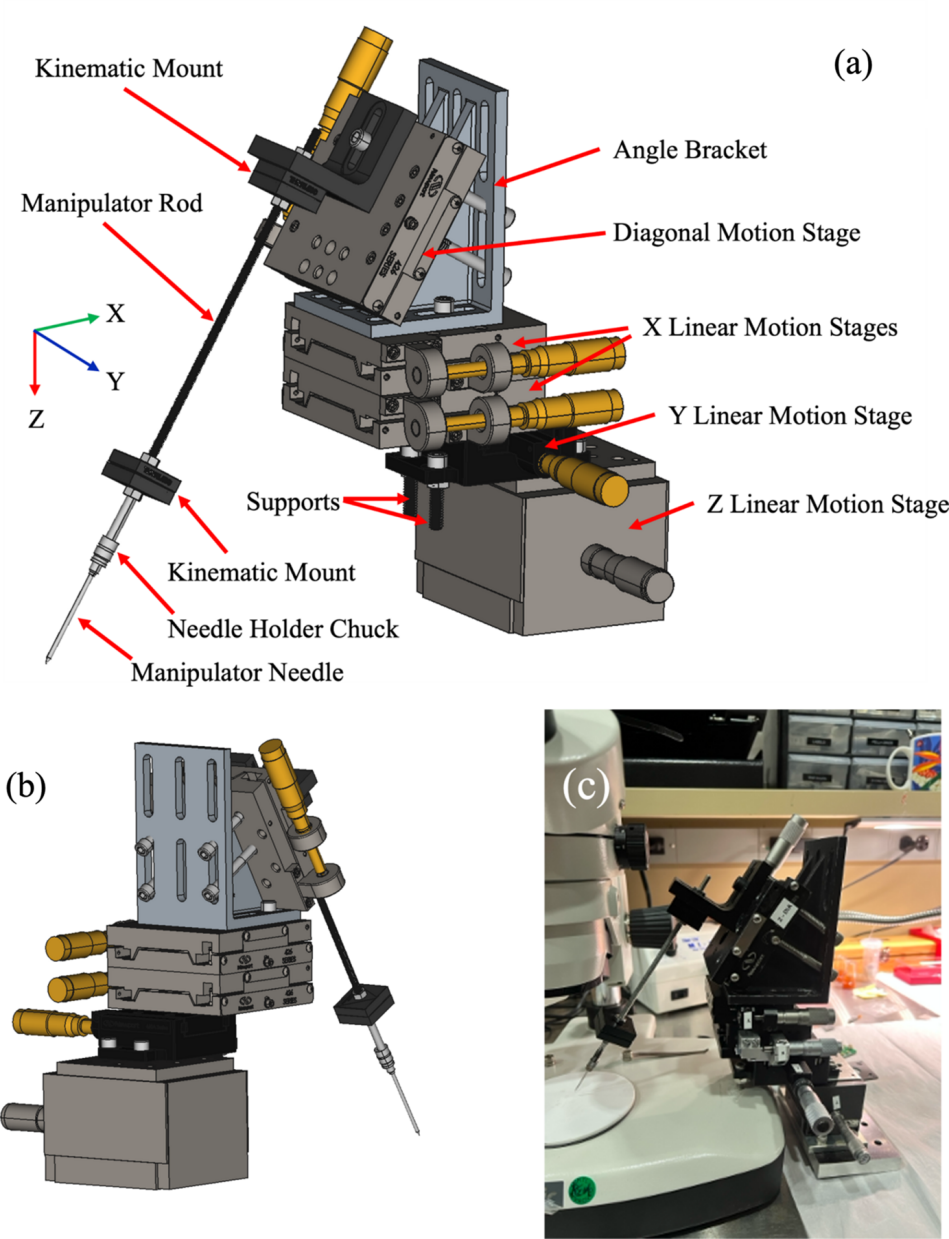
Sample manipulator for X-ray nanotomography samples. (*a*) Schematic design from front view, (*b*) schematic design from back view and (*c*) photograph of the manipulator in use with optical microscope.

The basic principle for the design of the manipulator is the need for the manipulator needle to move in a total of four directions. The *x*- and *y*-axes (perpendicular to each other) correspond to the *x* and *y* positions of the sample in the imaging plane of the optical microscope. The *z*-axis corresponds to the height of the manipulator needle from the sample, and a diagonal axis corresponds to the ∼60° angle at which the manipulator needle is placed and is used for bringing the needle closer and away from the sample. Motion in each of the axes is manually controlled by a vernier micrometre driving a linear motion stage. The *z* linear motion stage forms the base of the manipulator, and all the other components of the manipulator system rest on this stage as shown in Fig. 1 ▸(*a*). The reasoning behind such a design is that the *z* stage with its 13 mm of travel (Newport Corporation, model #MNV80) is used as the final step of either picking up a sample or attaching it to the tip of a vertically mounted mounting pin. Manual linear motion *x* and *y* stages with one- inch travel sit on top of the *z* stage. To gain reach into the microscope FOV we need a 50 mm range along the *x*-axis on the imaging plane. To achieve this, two 25 mm stages are stacked on top of each other. A 60° bracket supports the final 25 mm travel stage (diagonal axis stage) that drives the manipulator rod and needle into the FOV of the optical microscope from above and to the side while avoiding the microscope objective. To aid ease of mounting the manipulator rod and needle, the manipulator rod is attached to a small 90° angle bracket with a magnetic kinematic base pair (Thorlabs KB1X1). At the other end of the manipulator rod is another magnetic kinematic base pair that holds a small drill chuck and the manipulator hypodermic needle that is used to pick up and mount sample particles. The drill chuck that holds the needle provides flexibility to use a range of needle shapes and sizes. It also makes replacing the needle easy as frequent use results in blunting the needle tip with subsequent loss of precision. Using kinematic base plates to magnetically attach the drill chuck to the manipulator rod makes it easy and repeatable to remove the drill chuck while replacing the needle. Similarly, magnetically attaching the manipulator rod to the diagonal stage facilitates the easy removal of the manipulator rod with needle when the sample manipulator is not in use and the optical microscope is being used for general use.

### 2.2. Sample manipulator – operation

To perform nanotomography on samples that are in the form of small particles it is necessary to select a single particle that fits well within the FOV. Initially multiple particles of the sample are placed under an optical microscope and a <100 µm size particle is selected assuming the FOV of the nanotomography to be 100 µm × 100 µm. To scan this particle, it needs to be picked up from among other such particles and then attached to the tip of the sample mounting pin.

**Figure 2:**
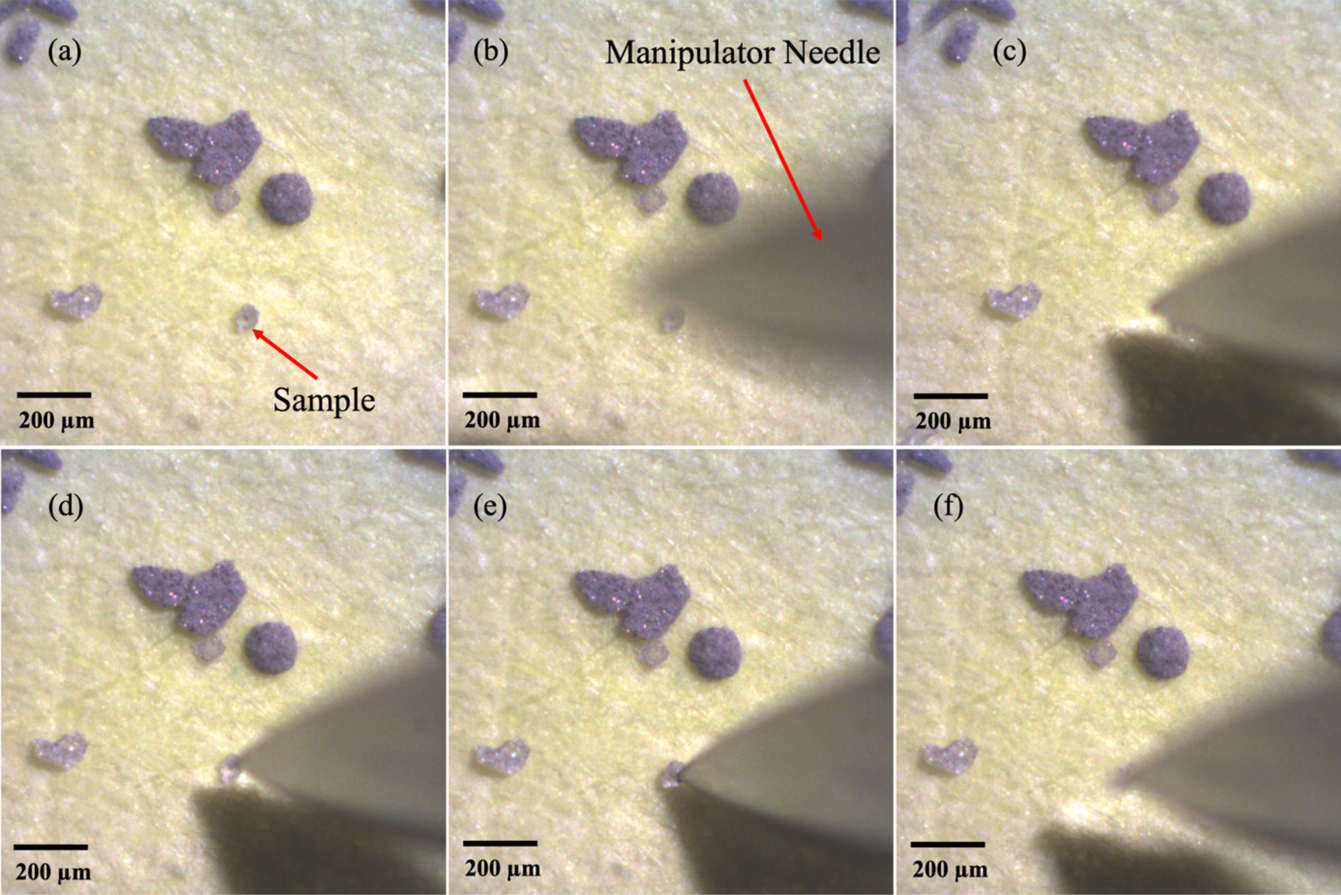
Optical image of the (*a*) nanotomography sample, (*b*) out-of-focus needle above the sample plane, (*c*) needle positioned just above the sample, (*d*, *e*) positioning needle tip at the sample and (*f*) needle raised with sample pick up.

To pick up a ∼50 µm glass bead, identified in Fig. 2 ▸(*a*), the sample manipulator is placed to the side of the optical microscope as shown in Fig. 1 ▸(*c*). By means of the *x*, *y* and diagonal axes the manipulator needle is moved into the FOV of the optical microscope and above the sample as shown in Fig. 2 ▸(*b*). Once the position of the needle is in the FOV it is moved closer to the sample by moving it in the diagonal axis until the tip of the needle is ∼500 µm away from the sample, as shown in Fig. 2 ▸(*c*). Although the diagonal axis moves the needle closer to the sample it is quite difficult to accurately make contact with the sample using this stage axis. As the needle moves in the diagonal axis the *x* and *y* positions of the needle also need to be continuously changed. Thus once the tip of the needle is ∼500 µm above from the sample, its *x* and *y* positions are adjusted to align with the sample as shown in Fig. 2 ▸(*d*), and then the *z* axis is used to make contact with the sample. It is not mandatory to maintain a distance of ∼500 µm from the needle tip to the sample before moving the needle using the *z* axis to make contact with the sample. However, a distance of ∼500 µm has been found to require the least number of corrections on the other axes while moving the *z* axis. As <100 µm samples are lightweight, these often stick to the manipulator needle due to static charge. Alternatively, a light adhesive can be applied to the tip of the needle by scratching the surface of some cellophane tape. Once the sample sticks to the tip of the manipulator needle the height of the needle is raised by moving the *z* stage as shown in Fig. 2 ▸(*f*). The manipulator needle with the sample is then extracted out by moving the diagonal axis.

**Figure 3:**
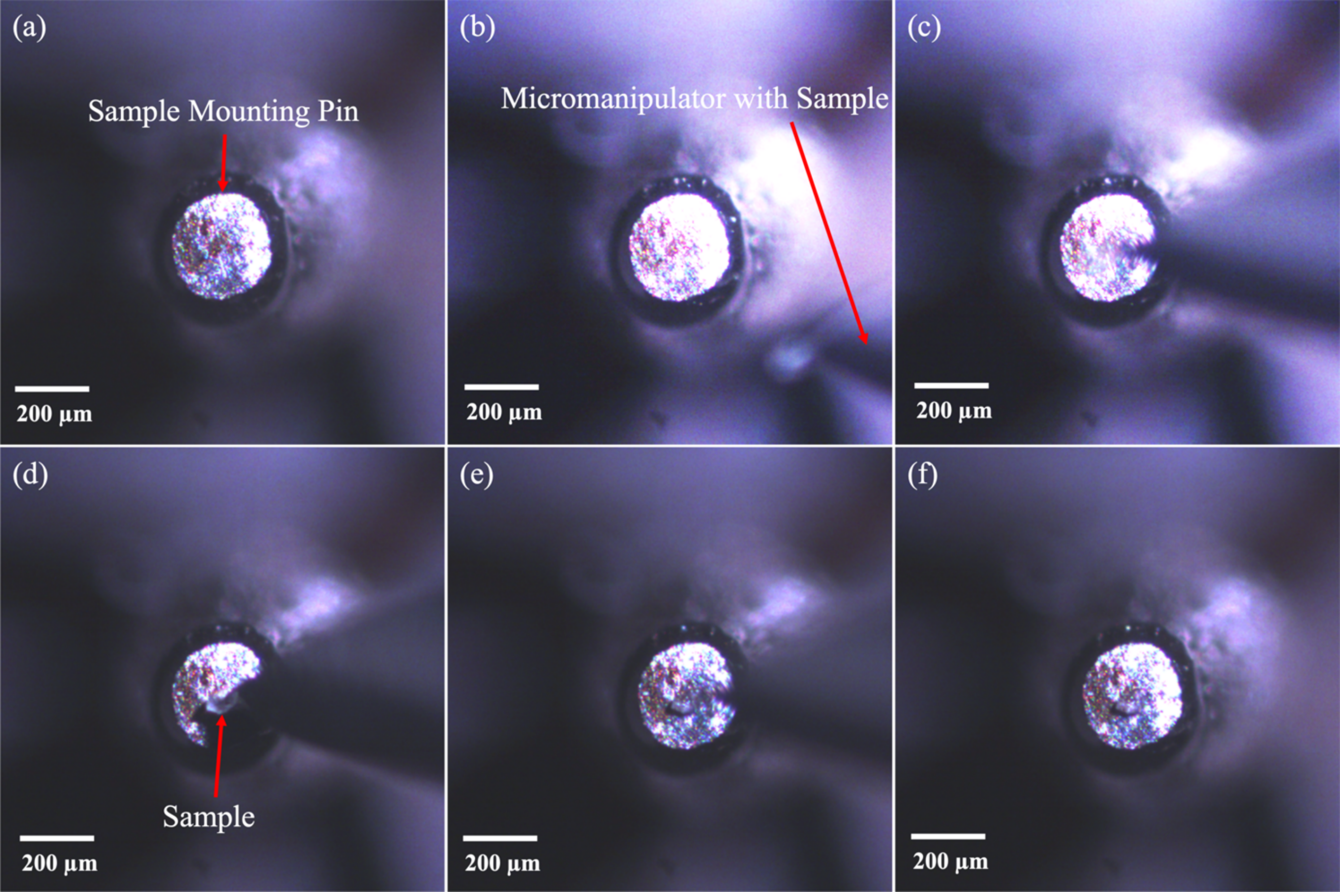
Optical image of the (*a*) ep­oxy coated flat end of sample mounting pin viewed end on. (*b*) 50 µm glass sphere sample plus manipular needle in the FOV but out of focus as above the mounting pin. (*c*) Align sample above pin. (*d*) Lower sample onto pin. (*e*) Sample sticks to pin and needle raised. (*f*) Needle retracted leaving sample on pin.

To mount the sample on a pin, the pointed end of a regular push pin is made flat by rubbing against abrasive polishing paper of 250 and 800 grit. A very thin film of ep­oxy is applied on this newly flattened surface and the pin placed vertically under the optical microscope, as shown in Fig. 3 ▸(*a*). The manipulator needle with the glass ball sample is brought into the FOV but is above the pin and out of focus [Fig. 3 ▸(*b*)]. Adjustment of the *x* and *y* axes aligns the sample above the pin and the sample is aligned at the center or any other preferred location on the mounting pin [Fig. 3 ▸(*c*)], before lowering the sample with the *z* axis onto the pin head until it makes contact with the ep­oxy film on the tip of the pin [Fig. 3 ▸(*d*)]. Once the ep­oxy holds the sample in position the manipulator needle is moved up [Fig. 3 ▸(*e*)] using the *z* stage and out from the FOV using the diagonal stage [Fig. 3 ▸(*f*)]. If the ep­oxy on the tip of the mounting pin is unable to retain the sample as it is strongly stuck to the manipulator needle, the sample is kept in contact with the ep­oxy until the ep­oxy hardens and then the manipulator needle is removed.

## 3. X-ray nanotomography compression–tension cell

Compression and tension experiments are one of the most common types of *in situ*X-ray tomography experiments that are regularly performed at light sources around the world. While such experiments can be performed with relative ease at micro-computed tomography beamlines, performing tension or compression experiments at nanotomography beamlines can be challenging. Challenges in performing compression/tension experiments at nanotomography beamlines arise due to the high imaging resolution and smaller FOV. Also, depending on the design of the beamline, if the zone plate is close to the sample then not enough space might be available to fit compression/tension cells of bulky design. Within these constraints we have developed a compression–tension cell with fluid flow-through capability for performing *in situ* mechanical testing of nanotomography samples.

### 3.1. Compression–tension cell – description

The compression–tension cell comprises two main components: a triaxial stage for sample alignment and a load cell to measure the applied load. The dimensions of the entire setup are such that it completely fits within the 101 mm × 89 mm footprint of the BLK-4 kinematic base plate (Newport Corporation) that the load cell is mounted on. This ensures that none of the components can come into contact with the beamline optics during the 180° rotation of the sample stage. Having the cell mounted on the BLK-4 kinematic base plate allows the setup to be easily mounted on its mating plate that is located on the sample rotating stage.

**Figure 4:**
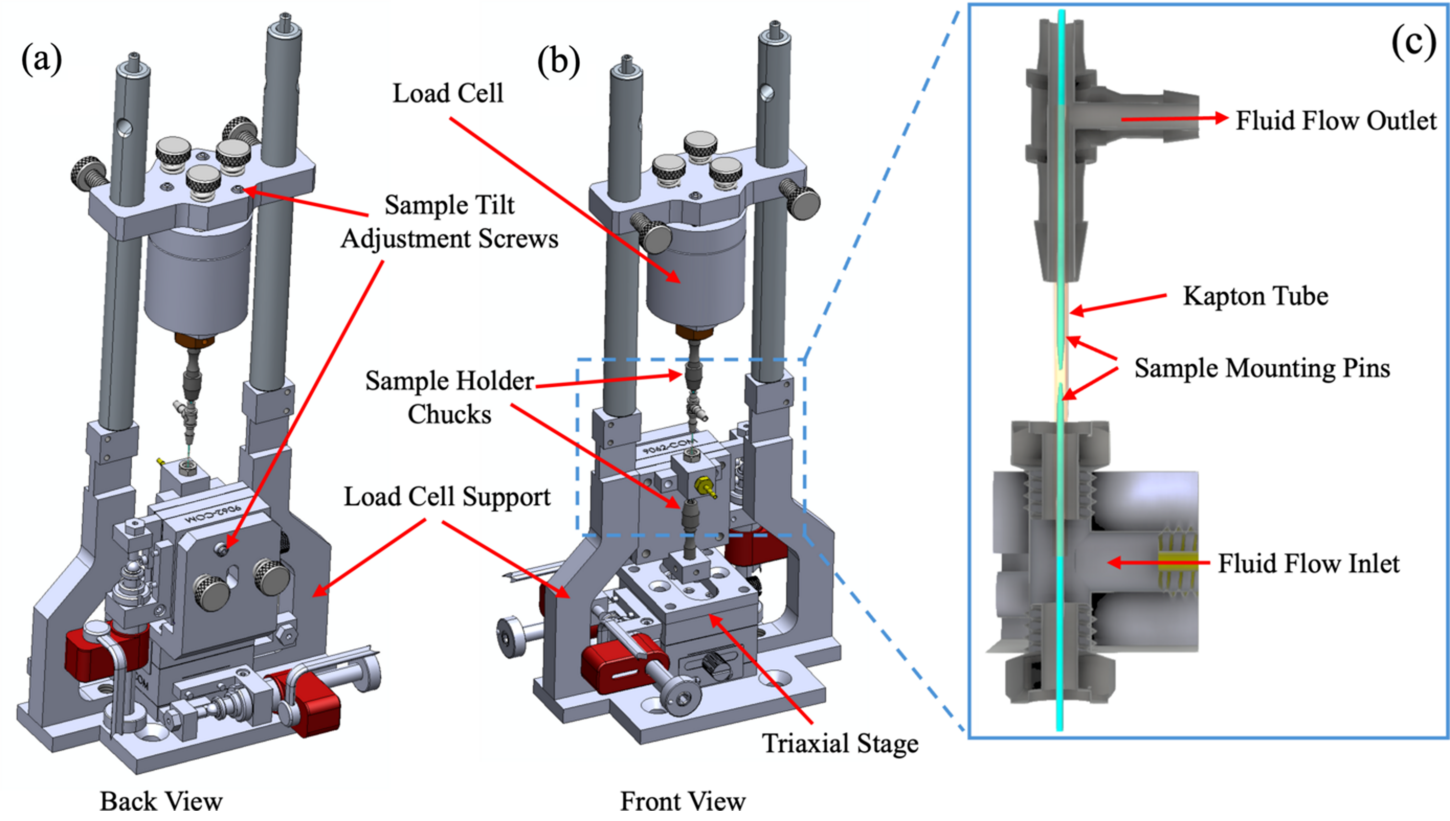
Schematic of the compression–tension cell. (*a*) Back view, (*b*) front view and (*c*) cross-sectional view of fluid flow system.

A motorized triaxial stage from Newport Corporation (9063-XYZ-PPP-M) is used as the sample stage for the compression–tension cell. The triaxial stage is fixed to the base plate of the cell which in turn is fixed to the BLK-4 base plate as shown in Figs. 4 ▸(*a*) and 4(*b*). The stage is moved along the *x*, *y* and *z* axes by three piezoelectric actuator motors with 25.4 mm travel that allows for aligning the sample within the nanotomography FOV. A small drill chuck is fixed to the stage for mounting the sample. Using a drill chuck provides the flexibility of using sample mounting pins of different shapes and sizes. The top half of the cell is a custom-built load cell from Novatech having a maximum load capacity of 5 N. Attached to this is a sample loading pin in a drill chuck in the inverted position. The load cell has bi-directional load measurement capability which allows for both tension and compression experiments. The Novatech load cell is supported above the lower triaxial stage by two vertical optical posts attached to the bottom base plate. The load cell supports are thinned to 4 mm over a length of 1.5 inch to reduce the number of sample projections lost as a result of the supports occluding the beam path during the 180° rotation of the sample stage during a tomography scan. As will be described below, samples can undergo compression and tension experiments under fluid flow conditions. It has been found to be very useful to mount the load cell in the upper section of the device as this avoids the load cell having to weigh the liquid when introduced to an empty cell. The liquid weight is of magnitude several mN which is comparable with the compression forces measured and so requires careful fluid control. This is avoided if the load cell is mounted in the top section of the cell.

For experiments involving compression of a single sample, the flat surface of the loading pin is used to apply the compressive force. For experiments analyzing the change at the interface of two samples as a result of the applied compressive force, both upper and lower sample mounting pins are loaded with samples. The Novatech load cell is fixed to the top base plate using three bolts with springs which allow for tilt adjustment. The base plate also contains three precision spring screws for tilt adjustment. Tilt adjustment is required if the sample pin flats are not orthogonal to the pin axis. This causes difficulty in sample alignment during the experiment and determining the center of rotation during reconstruction of the data.

For compression experiments involving fluid flow, a 37 mm-long stainless steel rod of ∼800 µm diameter is used as the sample loading pin. This long loading pin is attached to the triaxial stage. The pin is long enough to pass through a custom-built ‘tee’ coupling which seals around the base of the pin as shown in Fig. 4 ▸(*c*). A 1 mm inner diameter Kapton tube of 100 µm wall thickness is sealed to the top of the ‘tee’ coupling using silicon sealant leaving an annular gap of width ∼100 um around the sample pin through which fluid can flow. The bottom port of the ‘tee’ coupling through which the loading pin passes is also sealed using silicon sealant once the pin is in position. Fluid enters the ‘tee’ by means of the horizontal port of the ‘tee’ fitting. The upper sample pin has a similar sealing arrangement except vacuum grease is used to seal the opening of the Kapton tube after the top pin has been inserted. As such, the sample is surrounded by up to 1000 µm fluid within the 1 mm diameter Kapton tube.

### 3.2. Compression–tension cell – operation

For sample compression experiments, the sample particle is loaded on the sample loading pin using the sample manipulator as described in Section 2.2. The top and bottom pins are mounted on the triaxial stage and load cell sufficiently far apart that the samples do not touch prior to final alignment. It also needs to be ensured that the flat surfaces of both pins are within the thinned region of the load cell supports. Crude sample alignment to the millimetre range is achieved by eye with adjustment of the triaxial stage. Final sample alignment between top and bottom samples is achieved from orthogonal X-ray radiographic projection imaging of the sample. Ideally the sample is viewed from the 0°, 90° and 180° projections, but the 90° view is blocked by the load cell supports. An 80° viewing angle is found to be adequate for sample alignment. The thinned region of the load cell supports blocks the beam for ∼10° of the stage rotation. *Z* motion of the lower sample can bring the two samples into near contact. During the experiment 3D X-ray nanotomography scans are performed at different load steps. Load is applied by raising the lower sample on the triaxial stage.

## 4. Compression experiment with glass beads

For initial testing and to establish that the device is stable to allow *in situ* nanotomography experiments to be performed with the compression–tension cell, a compression experiment was performed with two glass beads in air. This requires precise aligning of the glass beads along the load axis. If the central axes of the beads are not precisely aligned, then instead of uniaxial compression the beads would easily shear against one another. As the mechanical properties of glass are known, a theoretical analysis can be compared with the experimental results.

To perform the experiment two ∼50 µm glass beads were loaded on the sample pins following the description given in Section 2.2. The sample pins were then loaded onto the mechanical cell as described in Section 3.2. To acquire nanotomography data of the uniaxial compression of the glass beads the mechanical cell with the glass beads was placed on the rotating sample stage at the 11.3.1 tender X-ray nano­tomography beamline at the Advanced Light Source (Nichols *et al.*, 2022 ▸). The sample along with the compression cell was allowed to stabilize for ∼30 min after closing the hutch door. Starting a scan immediately after placing a sample in the hutch leads to motion artifacts in the reconstructed data due to sample drift as the sample moves about 100 pixels during this period. Beyond 30 min sample drift is below the resolution limit of the radiograph and does not affect the reconstructed data. The beamline operates a transmission X-ray microscope in the energy range 5–17 K eV as selected by the channel-cut Si(111) monochromator. A monochromatic beam of 6.8 keV was used to image the glass beads during the compression experiment. The nanotomography beamline, as described by Nichols *et al.* (2022 ▸), illuminates the sample with two-pole illumination. It has since been upgraded to four-pole sample illumination with the addition of another pair of mirrors in the condenser section. This improves the fidelity of the imaging. Alignment of the two spheres was achieved as described in Section 3.2. Before applying load and compressing the glass beads an initial scan was performed without the beads being in contact. To begin the experiment a load of 69 mN was applied on the top bead by moving the bottom bead along the *z* axis on the triaxial stage by a displacement of 4 µm. Two more loading steps were performed each with 4 µm displacement that resulted in 139 mN load for the second step and the bottom bead fractured at the third loading step. 600 projections with 8 s exposures were collected for each scan. The pixel size of the radiographs is 43 nm.

Reconstruction of the nanotomography data was performed using a modified algorithm based on *TomoPy* (Gürsoy *et al.*, 2014 ▸). The NERSC supercomputing facility was used for the full reconstruction of the data. Projections where the beam was blocked by the load cell supports were not used during reconstruction. Including these projections in reconstruction of the data results in line artifacts. 3D visualization of the data was performed using *Dragonfly 2022.2* (Dragonfly ORS software; Dragonfly, undated ▸) and distance measurements were made using *ImageJ* (Schindelin *et al.*, 2012 ▸).

### 4.1. Results and discussion

**Figure 5:**
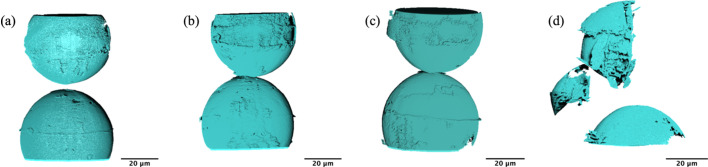
3D visualization of the glass beads during compression experiment at (*a*) initial separation, (*b*) 69 mN load, (*c*) 139 mN load and (*d*) fracture.

At the beginning of the experiment the glass beads are separated by ∼5 µm as shown in Fig. 5 ▸(*a*). The alignment of the beads needs to be such that the apexes of both the beads are aligned. Once the beads are compressed against one another with a load of 69 mN the apexes are no longer clearly visible in the visualization in Fig. 5 ▸(*b*). However this forms a contact area. By analyzing the reconstructed slices the contact area of the beads can be extracted. The load was increased to 139 mN by a 4 µm step size as shown in Fig. 5 ▸(*c*). Further increasing the load by a displacement of another 4 µm, the bottom bead fractures as shown in Fig. 5 ▸(*d*). Note that the images are inverted top to bottom. Reconstructed tomograms for each step are shown in Fig. S1 of the supporting information.

Although 4 µm displacement is applied at each load step the actual deformation of the glass beads is much less and not fully observable in the nanotomography data. However, from the reconstructed slices the actual contact area of the beads for the two intermediate loading steps can be determined and compared with analytical solutions of contact areas for spherical geometries under loads.

#### 4.1.1. Hertz analysis of contact area

When two bodies of spherical geometry are in contact under load their contact area is in the form of a circle which can be calculated using the Hertz equation (Hertz, 1881 ▸),
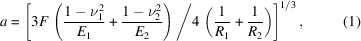
where *a* is the radius of the contact area of the spheres. *E*, ν and *R* are the Young’s modulus, Poisson’s ratio and radius of the spheres, respectively, and *F* is the compressive force. As the beads used in the experiment are made of glass, their Young’s modulus is 0.25 and Poisson’s ratio is 72 GPa. The radius of the top bead was estimated to be 25.75 µm and that of the bottom bead was estimated to be 23.75 µm from the reconstructed data.

Theoretical estimation of the contact area from the Hertz analysis results in a uniform circle. However, due to sample imperfections the actual contact area is seldom uniform or even a circle (Wang *et al.*, 2005 ▸; Persson *et al.*, 1993 ▸; Pastewka & Robbins, 2016 ▸). Two types of imperfections play a major role in the deviation of the actual contact area from the theoretical estimation. (1) Sphericity: the actual sample is never a perfect sphere and when a sample as small as ∼50 µm diameter is imaged with high resolution nanotomography small changes in sphericity can modify the contact area. This results in an experimental contact area which is larger or smaller than the theoretical estimation. (2) Surface roughness: the regions on the sphere that are higher will come in contact before the lower regions. This results in an experimental contact area that is not a circle.

**Figure 6:**
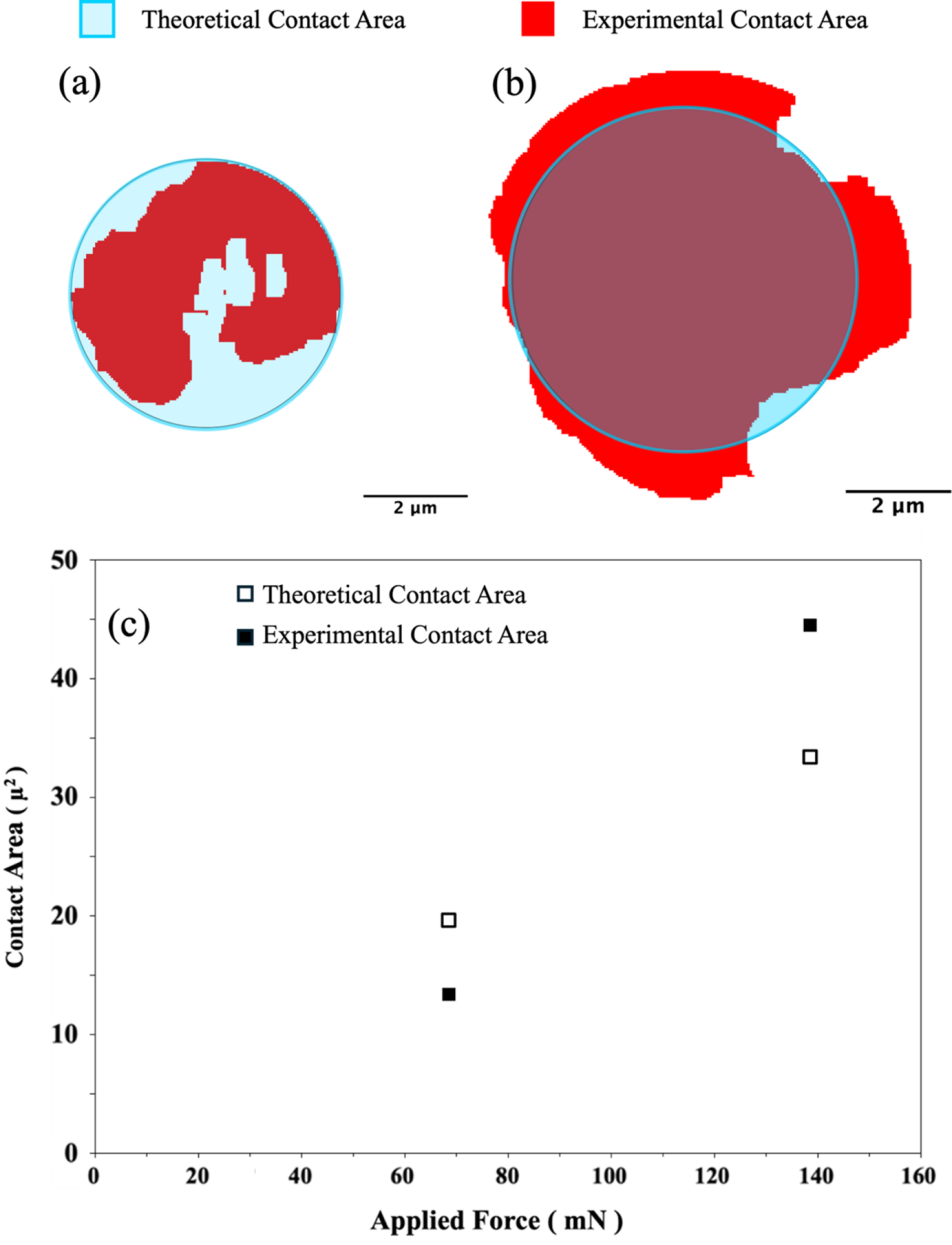
Comparison of experimental and theoretical contact areas for loads of (*a*) 69 mN and (*b*) 139 mN. (*c*) Plot showing the change in contact area with load.

The combined effect of these two imperfections is observed in the comparison of the experimental versus theoretical contact areas of the glass beads as shown in Figs. 6 ▸(*a*) and 6(*b*). The experimental contact area for a 69 mN applied compressive force is smaller than the theoretical estimate. Also, the experimental area in not a circle primarily due to the surface roughness of the beads. When the applied load is increased to 139 mN the situation reverses and the experimental contact area becomes slightly larger than the theoretical estimate and its shape is much closer to that of an actual circle. As the load increases, the lower regions on the contact surface of the beads also come into contact thus the contact area comes closer to having a circular geometry. However, imperfection in sphericity of the beads makes the contact area larger than the theoretical estimate.

## 5. Compression experiment with fluid flow

To demonstrate *in situ* fluid flow capabilities of the cell design we describe a compression experiment with fluid flow with two calcite grains when compressed against each other under a calcite saturated solution surrounding the two grains.

Initially the calcite grains were brought close to each other and an initial tomographic scan was performed at 6800 eV. The grains were then compressed against each other with a force of 10 mN while flowing the calcite saturated solution. The calcite grains were kept in constant load for 12 h and then separated for scanning. After completion of the scan the grains were again compressed with a load of 40 mN and kept in constant load for another 12 h after which they were separated and scanned.

**Figure 7:**
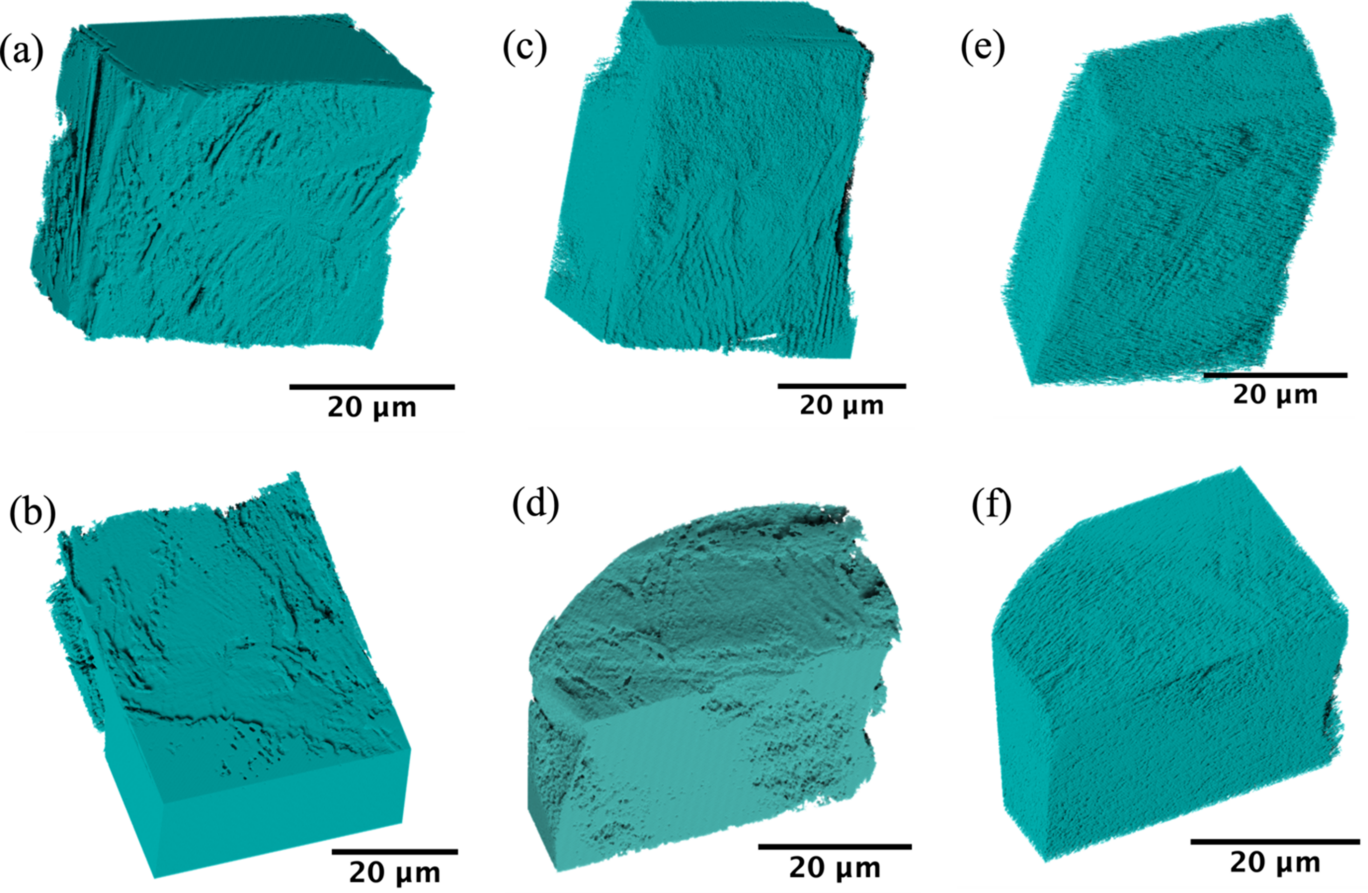
3D visualization of calcite. (*a*) Initial top grain, (*b*) initial bottom, (*c*) top grain after 12 h at 10 mN, (*d*) bottom grain after 12 h at 10 mN, (*e*) top grain after 12 h at 40 mN and (*f*) bottom grain after 12 h at 40 mN.

Fig. 7 ▸ shows the 3D visualization of the reconstructed data for the calcite grains in the region where the grains are in contact. Figs. 7 ▸(*a*) and 7(*b*) are the initial top and bottom grains. They have been reorientated to allow for easier viewing of the contacting surfaces. Figs. 7 ▸(*c*)–7(*f*) show a significant change in surface roughness as the experiment progressed. The surface features of the calcite grains become much smoother with load and time. It is postulated that this is due to dissolution and precipitation of calcite under pressure in a calcite saturated solution. During the experiment some regions of the bottom grain moved outside the field of view as a result of shearing between the grains after initial contact. This results in the shape of the bottom grains in Figs. 7 ▸(*d*) and 7(*f*) appearing different from those in Fig. 7(*b*). A more detailed analysis, such as r.m.s. roughness measurements, mass movement determination and rate dissolution estimates from these measurements is beyond the scope of this paper.

## 6. Conclusion

A compression–tension cell with sample manipulator has been built for conducting *in situ* mechanical experiments at X-ray nanotomography beamlines. Samples can also be in a fluid flow environment. A specialized sample manipulator was built for mounting small (<100 µm) samples on sample pins that can be installed in the nanotomography compression–tension cell.

The compression–tension cell consists of a triaxial stage for aligning the sample in the *x* and *y* directions and applying sample force in the *z* direction. Applied load is measured by a custom built bi-directional load cell. The device also has the capability for fluid flow-through for conducting compression–tension experiments in a fluid environment. Precision screws at the base of the triaxial stage and that of the load cell helps with tilt correction of samples.

A compression experiment where two glass beads were compressed with uniaxial compressive force was conducted using the mechanical cell. During the experiment load was applied from zero load condition until one of the glass beads fractured. Nanotomography scans were performed at each load step. The instrument proved to be adequately stable and the experimental observation of the contact area of the glass beads was compared with the theoretical estimation of the contact area using the Hertz analysis. To demonstrate the fluid flow capabilities of the device, two calcite grains were compressed together in a calcite saturated solution. After 24 h and 10–40 mN pressure the calcite surfaces under contact reduced their surface roughness probably due to dissolution and precipitation of calcite under pressure.

These experiments demonstrate the highly precise design of the sample manipulator and the compression–tension cell for *in situ* nanotomography mechanical experiments such as compression/tension experiments to study the nano-mechanical behavior of samples and corrosion studies under load conditions in the field of material science as well as chemical–mechanical studies of geological samples under fluid flow conditions. As such these designs can be used for construction of similar devices for laboratory based X-ray tomography systems and synchrotron beamlines around the world.

## Supplementary Material

Supplementary Figure S1. DOI: 10.1107/S1600577525005053/ok5141sup1.pdf

## References

[bb1] Antonelli, S., Ronne, A., Han, I., Ge, M., Layne, B., Shahani, A. J., Iwamatsu, K., Wishart, J. F., Hulbert, S. L., Lee, W.-K., Chen-Wiegart, Y.-K. & Xiao, X. (2020). *J. Synchrotron Rad.* **27**, 746–752. 10.1107/S1600577520004567 PMC7285687 32381777

[bb2] Barnard, H. S., Macdowell, A. A., Parkinson, D. Y., Larson, N. M., Peterson, J. C., Panerai, F., Mansour, N. N. & Gao, Y. (2018). *Microsc. Microanal.* **24**, 444–445.

[bb3] Bhattacharjee, A. J., Yost, A. R., Erdeniz, D., Dunand, D. C. & Paz y Puente, A. E. (2021). *Intermetallics* **134**, 107199.

[bb101] Dragonfly (undated). *Dragonfly 2022.2.* Comet Technologies Canada Inc., Montreal, Canada; software available at https://www.theobjects.com/dragonfly.

[bb5] Engelkes, K., Friedrich, F., Hammel, J. U. & Haas, A. (2018). *Zoomorphology* **137**, 213–228.

[bb6] Gürsoy, D., De Carlo, F., Xiao, X. & Jacobsen, C. (2014). *J. Synchrotron Rad.* **21**, 1188–1193. 10.1107/S1600577514013939 PMC4181643 25178011

[bb7] Hertz, H. (1881). *J. Reine Angew. Math.* **96**, 156.

[bb8] Kaira, C. S., Stannard, T. J., De Andrade, V., De Carlo, F. & Chawla, N. (2019). *Acta Mater.* **176**, 242–249.

[bb9] Kelly, T. F. & Miller, M. K. (2007). *Rev. Sci. Instrum.* **78**, 031101. 10.1063/1.2709758 17411171

[bb10] Kudrna Prašek, M., Pistone, M., Baker, D. R., Sodini, N., Marinoni, N., Lanzafame, G. & Mancini, L. (2018). *J. Synchrotron Rad.* **25**, 1172–1181. 10.1107/S1600577518005970 29979179

[bb11] Liang, C., Wang, F., Fan, W., Zhou, W. & Tong, Y. (2017). *TrAC Trends Anal. Chem.* **90**, 62–79.

[bb12] Maire, E., Buffière, J. Y., Salvo, L., Blandin, J. J., Ludwig, W. & Létang, J. M. (2001). *Adv. Eng. Mater.* **3**, 539.

[bb13] Miller, M. K., Kelly, T. F., Rajan, K. & Ringer, S. P. (2012). *Mater. Today* **15**, 158–165.

[bb14] Müller, E. W., Panitz, J. A. & McLane, S. B. (1968). *Rev. Sci. Instrum.* **39**, 83–86.

[bb15] Nichols, J. B., Voltolini, M., Gilbert, B., MacDowell, A. A. & Czabaj, M. W. (2022). *Rev. Sci. Instrum.* **93**, 023704. 10.1063/5.0076322 35232135

[bb16] Pastewka, L. & Robbins, M. O. (2016). *Appl. Phys. Lett.* **108**, 221601.

[bb17] Persson, J., Breder, K. & Rowcliffe, D. J. (1993). *J. Mater. Sci.* **28**, 6484–6489.

[bb18] Philippe, J., Le Godec, Y., Mezouar, M., Berg, M., Bromiley, G., Bergame, F., Perrillat, J. P., Alvarez-Murga, M., Morand, M., Atwood, R., King, A. & Régnier, S. (2016). *High Pressure Res.* **36**, 512–532.

[bb19] Ruf, M., Lee, D. & Steeb, H. (2023). *Rev. Sci. Instrum.* **94**, 085115. 10.1063/5.0153042 38065175

[bb20] Schindelin, J., Arganda-Carreras, I., Frise, E., Kaynig, V., Longair, M., Pietzsch, T., Preibisch, S., Rueden, C., Saalfeld, S., Schmid, B., Tinevez, J.-Y., White, D. J., Hartenstein, V., Eliceiri, K., Tomancak, P. & Cardona, A. (2012). *Nat. Methods* **9**, 676–682. 10.1038/nmeth.2019 PMC3855844 22743772

[bb21] Voltolini, M., Barnard, H., Creux, P. & Ajo-Franklin, J. (2019). *J. Synchrotron Rad.* **26**, 238–243. 10.1107/S1600577518015606 30655491

[bb22] Wang, C. X., Cowen, C., Zhang, Z. & Thomas, C. R. (2005). *Chem. Eng. Sci.* **60**, 6649–6657.

[bb23] Williams, D. B. & Carter, C. B. (1996). *Transmission Electron Microscopy*, pp. 3–17. Boston: Springer US.

[bb24] Zhou, T., Babu, R. P., Hou, Z. & Hedström, P. (2022). *Crit. Rev. Solid State Mater. Sci.* **47**, 388–414.

