# Supplementary material for: Compression–tension cell with sample manipulator for in situ X-ray nanotomography experiments
Source: J Synchrotron Radiat. 2025 Jul 14;32(Pt 5):1282–90. doi: 10.1107/S1600577525005053 (PMC12416415; doi:10.1107/S1600577525005053)
Supplement: Supplementary file 1 [file s-32-01282-sup1.pdf]

JOURNAL OF SYNCHROTRON RADIATION

Volume 32 (2025)

Supporting information for article:

Compression-Tension Cell with Sample Manipulator for *In-situ* X-ray Nanotomography Experiments

Arun J. Bhattacharjee, Harold S. Barnard, Alastair MacDowell, Dilworth Parkinson and Harrison P. Lisabeth

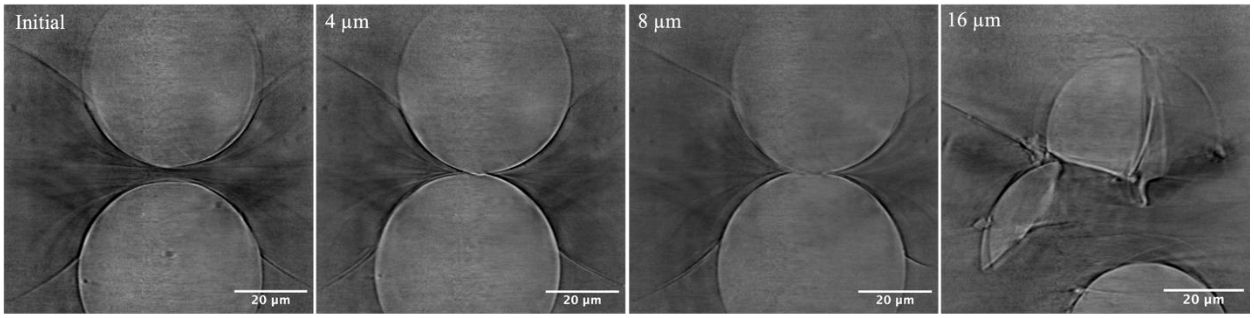

Figure S1 Reconstructed tomogram showing the glass beads before contact, after 4µm displacement, after 8 µm displacement and after 16 µm displacement.
